# In situ efficacy of an experimental toothpaste on enamel rehardening and prevention of demineralisation: a randomised, controlled trial

**DOI:** 10.1186/s12903-020-01081-y

**Published:** 2020-04-17

**Authors:** Jonathan E. Creeth, Gary R. Burnett, Audrey Souverain, Paola Gomez-Pereira, Domenick T. Zero, Frank Lippert, Anderson T. Hara

**Affiliations:** 1 GSK Consumer Healthcare, St George’s Avenue, Weybridge, Surrey, KT13 0DE UK; 2 GSK Consumer Healthcare, Route de l’Etraz 2, 1260 Nyon, Switzerland; 3 Department of Cariology, Operative Dentistry and Dental Public Health and Oral Health Research Institute, Indiana University School of Dentistry, 415 Lansing Street, Indianapolis, IN 46202 USA

**Keywords:** Sodium fluoride, Dentifrice, Erosion, Clinical study

## Abstract

**Background:**

A novel sodium fluoride toothpaste containing lactate ion and polyvinylmethylether-maleic anhydride has been developed to promote enamel remineralisation and resistance to demineralisation. In this in situ study, we compared this toothpaste (‘Test’) with a stannous fluoride-zinc citrate (SnF_2_-Zn) toothpaste (‘Reference’) (both 1100–1150 ppm fluoride) and a fluoride-free toothpaste (‘Placebo’) using an enamel dental erosion-rehardening model.

**Methods:**

In each phase of this randomised, investigator-blind, crossover study, participants wore palatal appliances holding bovine enamel specimens with erosive lesions. They brushed their natural teeth with either the Test, Reference or Placebo toothpastes, then swished the resultant slurry. Specimens were removed at 2 h and 4 h post-brushing and exposed to an in vitro acid challenge. Surface microhardness was measured at each stage; enamel fluoride uptake was measured after in situ rehardening. Surface microhardness recovery, relative erosion resistance, enamel fluoride uptake and acid resistance ratio were calculated at both timepoints.

**Results:**

Sixty two randomised participants completed the study. Test toothpaste treatment yielded significantly greater surface microhardness recovery, relative erosion resistance and enamel fluoride uptake values than either Reference or Placebo toothpastes after 2 and 4 h. The acid resistance ratio value for Test toothpaste was significantly greater than either of the other treatments after 2 h; after 4 h, it was significantly greater versus Placebo only. No treatment-related adverse events were reported.

**Conclusions:**

In this in situ model, the novel-formulation sodium fluoride toothpaste enhanced enamel rehardening and overall protection against demineralisation compared with a fluoride-free toothpaste and a marketed SnF_2_-Zn toothpaste.

**Trial registration:**

ClinicalTrials.gov; NCT03296072; registered September 28, 2017.

## Background

Erosive tooth wear develops as a consequence of intra-oral acid exposure from dietary and/or gastric sources. Such processes can cause enamel surfaces to soften and become more susceptible to abrasive wear or attrition, leading to progressive loss of dental hard tissue [1, 2]. In the initial stages of dental erosion, the enamel surface can be rehardened by calcium and phosphate ions naturally present in saliva, removing this susceptibility [3–5].

As well as having a role in preventing dental caries, fluoride-containing toothpastes promote enamel remineralisation after exposure to dietary acid, leaving a fluoridated surface that is more resistant to subsequent acid exposure [6–8]. This fluoride benefit has been observed as early as 1 h after toothpaste use in in situ experimental conditions similar to those used here, and seen to progressively increase after 2 and 4 h [9]. Previous studies have tested the hardness of the enamel surface using the ‘surface microhardness test’ to detect changes in mineral content [6–10].

The most common fluoride sources for modern toothpastes are sodium fluoride (NaF) and stannous fluoride (SnF_2_), with some studies suggesting that stabilised SnF_2_ offers greater protection against dental erosion than other fluoride compounds [11–14]. The formulation of a toothpaste has been shown to have the potential to influence fluoride’s ability to protect against dental erosion; in situ studies have indicated that some non-fluoride ingredients in a toothpaste formulation, such as zinc ions, sodium hexametaphosphate and sodium phytate, may modulate the effects of fluoride on remineralisation–demineralisation [6, 15–17]. Previously, optimisation of the enamel protection ability of fluoride ion has been achieved by omitting ingredients that either bind directly to fluoride or interfere with fluoride binding to enamel [18]. The Test toothpaste in this study builds on this approach by also including polyvinylmethylether-maleic anhydride (PVM/MA) copolymer, observed to increase fluoride’s ability to enhance acid resistance in vitro. It also included sodium lactate at a pH controlled to 6.2, observed to enhance enamel fluoride uptake (EFU) in vitro compared to a matched formulation at near-neutral pH without lactate (data on file).

In this in situ clinical study, we aimed to determine the ability of the Test toothpaste to enhance rehardening (2 and 4 h after toothpaste treatment) of enamel previously softened with dietary acid; to promote fluoride uptake to that surface; and to inhibit subsequent demineralisation of the rehardened surface. The effects of this formulation were compared to those of a fluoride-free (Placebo) toothpaste and a Reference toothpaste containing 1100 ppm fluoride as SnF_2_ plus zinc citrate.

## Methods

We conducted a single-centre, randomised (to order in which toothpastes were used), oral/dental examiner- and specimen analyst-blind, three-period, three-treatment, in-situ crossover study at the Indiana University Oral Health Research Institute. The Indiana University Institutional Review Board (IRB #1709160589) approved the protocol; the study was designed according to CONSORT guidelines and was conducted in accordance with the Declaration of Helsinki, the International Conference on Harmonisation of Technical Requirements for Registration of Pharmaceuticals for Human Use and local laws and regulations. As the study involved human participants, it was registered at clinicaltrials.gov (#NCT03296072). There was one administrative change to the protocol prior to study start that did not affect study flow or outcomes. Anonymised individual participant data and study documents can be requested for further research from www.clinicalstudydatarequest.com.

### Experimental design: overview

**Fig. 1:**
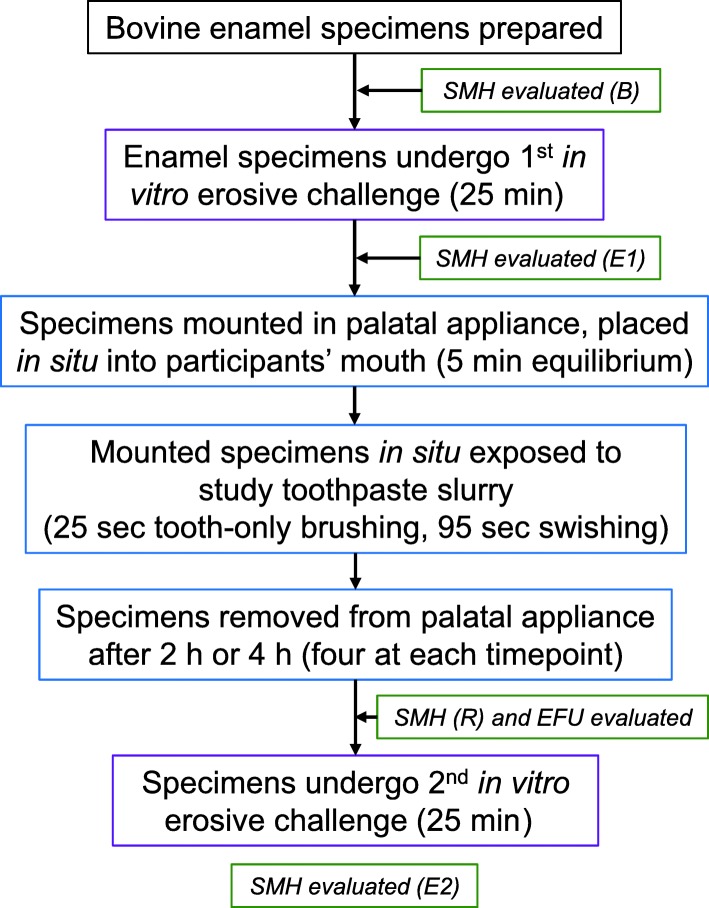
Study design

This study utilised an in situ dental erosion–remineralisation model developed by Zero et al., [8] that has been used in a number of similar studies [6–8, 15–17]. In brief (Fig. 1), specimens of bovine enamel firstly underwent an erosive challenge in vitro, then were attached to a palatal appliance that was worn by a study participant, and the assigned toothpaste was used as directed. The enamel specimens were removed from the palatal appliance at 2 and 4 h post-toothpaste exposure for laboratory assessment by the specimen analyst that included a second in vitro erosive challenge. Further details of each step are provided below.

### Participants

Participants were recruited from an existing panel of individuals who had been pre-fitted with a maxillary palatal appliance capable of housing prepared enamel specimens. All participants were from the Indianapolis, IN area, where community water contains approximately 0.75 ppm fluoride [19]. Eligible participants were aged 18 to 65 years, in good general and oral health and had unstimulated/stimulated salivary flow rates of ≥0.2 mL/min and ≥ 0.8 mL/min, respectively. Exclusion criteria included: presence of cavitated carious lesions (determined by visual assessment only), moderate or severe periodontal conditions, or severe tooth wear; wearing an oral appliance or orthodontia (except permanent lower retainers); any condition that might have influenced the study or impacted participant safety and wellbeing; pregnancy; breastfeeding; use of any medication that could interfere significantly with salivary flow; an intolerance/hypersensitivity to study materials; use of any investigational products or participation in another clinical trial within 30 days of screening.

### Enamel specimen preparation

Bovine enamel specimens (1488 in total), taken from the central area of the buccal surface of lower incisors (up to two specimens per tooth), were ground-polished flat until the enamel surfaces had a minimum 3 mm × 3 mm facet in the centre. Specimens were then serially polished with grit papers of descending coarseness, finished with a polishing cloth wetted with a 1 μm diamond suspension then sonicated to remove any adherent polishing particles. The resulting specimens had a thickness range of 1.7–2.2 mm.

Changes in the mineral content of the enamel specimens during the experiment were evaluated using indentation to determine surface microhardness (SMH) [8, 20, 21]. At baseline, five indentations 100 μm apart were made in the centre of each enamel specimen using a Knoop diamond under a 50 g load, applied for 11 s. Analysis of indent lengths was performed on a specimen level and then averaged, with a participant-level average value used for analysis.

For the first in vitro erosive challenge, enamel specimens were demineralised by immersion in 35 ± 1 mL grapefruit juice (100% juice, Kroger Co, Cincinnati, OH, USA; pH range 2.8–3.1 over the different study days) with no stirring for 25 min, then thoroughly rinsed with deionised water. This time period was chosen as previous studies have shown this to be an adequate amount of time in demineralising conditions for the surface microhardness test to detect changes without significant surface loss (in the absence of agitation) [22]. Indentation lengths were then determined as before.

Following sterilisation with ethylene oxide, demineralised enamel specimens were secured onto plastic holders and attached to the palatal appliance, four on each side. Following the in situ challenge period (details below), after removal from the appliance, the specimens underwent a second in vitro erosive challenge as above (Fig. 1).

### In situ procedure

Participants were required to complete four study visits: screening (Visit 1), then three treatment visits (Visits 2, 3, and 4) at which each of the three treatments were evaluated in a crossover manner. Each treatment visit was separated by a washout period of at least 3 days that included at least 1 day’s use of the participant’s regular toothpaste and 2 days' use of a non-fluoridated (non-marketed) toothpaste immediately prior to the visit using a provided toothbrush (Oral-B® Sensi Soft Manual; Procter & Gamble Company, Cincinnati, OH, USA). At Visit 1, participants gave written informed consent to take part in the study and their demographics, medical history and prior medications were recorded. Oral hard tissue (OHT) and oral soft tissue (OST) examinations were performed, followed by saliva flow rate assessment. Eligible participants had their palatal appliance assessed for comfort, with new appliances made for those whose appliance no longer fitted adequately.

At Visits 2, 3, and 4, participants underwent a pre-treatment OST examination, then the study investigator (the oral/dental examiner) placed the palatal appliance holding the eight pre-demineralised bovine tooth enamel specimens in the participant’s mouth. An equilibration period of at least 5 min was given to allow for development of a salivary pellicle on the enamel blocks.

Participants received the study treatments detailed in Table 1 in a predetermined order according to a randomisation schedule generated by a contracted statistical analysis organisation using a Williams Square design balanced for first period carryover. Study numbers were allocated in ascending order as each participant was entered into the trial.

**Table 1:** Study treatments

Toothpaste group	Relevant formulation ingredients
Test toothpaste	0.254% w/w NaF (1150 ppm fluoride), 5% KNO_3_, PVM/MA copolymer, sodium lactate, pH 6.2
Placebo toothpaste	5% KNO_3_, PVM/MA copolymer, sodium lactate (0 ppm fluoride)
Reference toothpaste^a^	0.454% w/w SnF_2_ (1100 ppm fluoride), zinc citrate (‘SnF_2_-Zn’)

^a^Crest® Pro-Health Sensitivity and Enamel Shield (Smooth Formula); Procter & Gamble Company, Cincinnati, OH, USA; US-marketed product

The oral/dental examiner and the specimen analyst were not permitted in the room where study products were dispensed. The oral/dental examiner, specimen analyst, study statistician and any relevant study sponsor or research centre employees were blinded to treatment received. While treatment group was not revealed to the participant during the study and the study toothpastes were supplied to the study site in over-wrapped tubes to conceal product identity, participants could not be deemed fully blinded as, according to ICH guidelines, for a truly double blind study, the products would need to be identical in every way, including taste and texture, which they were not.

At each study visit, following the equilibration period, the participant was provided with a new toothbrush (as above) loaded with 1.5 g of the assigned toothpaste. The participant brushed the buccal surfaces of their natural teeth only for 25 timed seconds and then swished the resulting toothpaste slurry around their mouth for 95 s. After expectorating the slurry, the participant gently rinsed their mouth with 15 mL tap water for 10 s before expectorating again.

After completing the brushing/rinsing procedures, participants continued to wear their palatal appliance for a total of 4 h. Enamel specimens were removed in a predetermined order from the appliance at 2 and 4 h (four at each timepoint). Once the appliance was removed from the participant’s mouth after 4 h, a post-treatment OST examination was performed.

### Safety

Adverse events (AEs) and any abnormalities in the OHT or OST examinations were recorded from the end of screening until 5 days after the last administration of study product. Clinical judgement was exercised by the oral/dental examiner to diagnose the AE and to assess the relationship between the study product and occurrence of each AE, with intensity graded as mild, moderate, or severe.

### Specimen analysis

Indentation lengths (details above) were determined prior to (B) and after (E1) the first in vitro erosive challenge, after the treatment-induced in situ rehardening phase (R), and after the second in vitro erosive challenge (E2) (Fig. 1). The extent of rehardening was calculated as SMH recovery (SMHR) where %SMHR = {(E1-R)/(E1-B)}*100 [23]. Overall resistance of treated enamel to the erosive challenge was calculated as relative erosion resistance (RER), where %RER = {(E1-E2)/(E1-B)}*100 [24]. Acid resistance following intra-oral rehardening after treatment with the study toothpastes was calculated as the acid resistance ratio (ARR) where ARR = 1-{(E2-R)/(E1-B)} [7].

EFU was assessed by microdrill enamel biopsy after the in situ rehardening period, before the second erosive challenge [25]. Enamel specimens were drilled to a depth of 100 μm through the entire lesion, with four cores obtained per specimen. The enamel powder sample pooled from the four cores was dissolved in perchloric acid (20 μl of 0.5 M HClO_4_) then buffered with a citrate/ethylenediaminetetraacetic acid solution prior to analysis via a pre-calibrated fluoride-specific electrode. The diameter of the drilled cores was measured via light microscopy and the EFU expressed as amount of fluoride divided by the combined area of the enamel cores (μg F/cm^2^). These values were averaged across the four enamel specimens evaluated at each timepoint to produce the participant-wise mean EFU.

### Statistical analysis

Sufficient participants were screened so that up to 66 could be randomised to treatment, aiming to ensure that 60 evaluable participants completed the study. This sample was calculated to have 90% power to detect a difference in mean %SMHR of 5.0 between study products at 4 h, assuming a standard deviation (SD) of differences of 11.92 (from a previous, unpublished study, data on file), using a paired t-test with a 0.05 two-sided significance level. This sample size was calculated to have 80% power to detect a difference in %RER of 7.4 between study products. While these specific calculations were based on an unpublished study, participant numbers are similar to or higher than a number of previous in situ erosion-remineralisation model studies [6, 7, 11–13, 16, 20].

Efficacy analyses were conducted on a modified intent-to-treat (mITT) population, defined as all participants who were randomised into the study, received at least one dose of study product and had at least one post-baseline efficacy assessment. The safety population included all randomised participants who received at least one dose of study product.

The primary efficacy endpoint was the difference between the Test and Placebo toothpastes in %SMHR after 4 h of intraoral exposure. The difference was required to be statistically significant (*p* < 0.05) to meet the success criteria of the study. Secondary efficacy endpoints included the differences in %RER and EFU between the Test and Placebo toothpastes and in all measures between the Test and Reference toothpastes after 4 h. Exploratory endpoints included the difference in all measures for all paired efficacy comparisons after 2 h. *Post-hoc* analyses were comparison of ARR values between all treatments at both timepoints, and all other paired efficacy comparisons for each endpoint between the Placebo and Reference toothpastes at both timepoints.

Statistical analyses for all endpoints were performed using a mixed model analysis of variance (ANOVA) model that included fixed factors for study period and treatment and a random effect for participant. Statistical testing of all endpoints in this study was conducted at a two-sided significance level of 0.05. As a primary objective was defined prior to analysis, there was no adjustment for multiple comparisons. Adjusted means of all treatments and treatment differences were provided together with their standard error (SE), 95% confidence intervals (CIs) and *p-*values.

## Results

The first participant was enrolled on November 13, 2017; the last participant completed the study on January 16, 2018. Of the 68 participants screened, 62 were randomised to treatment and completed the study. All randomised participants were included in the mITT and safety populations. The majority of participants were female (*n* = 45; 72.6%) and were of White/Caucasian/European heritage (*n* = 42; 67.7%), with 14 (22.6%) of African American/African heritage, and six (9.7%) of Asian heritage or multiple races. Overall mean age was 43.4 (SD 13.38) years, with a range of 20 to 65 years.

### Efficacy

Enamel microhardness mean indentation lengths as a function of treatment, stage of the experiment and duration of rehardening are shown in Table 2. Figures for each efficacy endpoint (%SMHR, %RER, EFU, ARR) reflect raw mean values following the respective analysis detailed in the Methods section. Table 3 details the difference between the adjusted mean values (from the ANOVA model) of each toothpaste group and provides statistical analysis regarding the significance of these differences.

**Table 2:** Enamel microhardness mean indentation lengths [μm (±SE)] (mITT population, *n* = 62)

Time point	Treatment	Baseline [B]	After first demineralisation [E1]	After intraoral exposure [R]	After second demineralisation [E2]
2 h	Test	43.4 (0.1)	59.7 (0.2)	55.8 (0.3)	64.8 (0.3)
	Reference	43.4 (0.1)	59.8 (0.1)	56.5 (0.2)	66.9 (0.3)
	Placebo	43.4 (0.1)	60.0 (0.2)	56.9 (0.2)	71.0 (0.5)
4 h	Test	43.3 (0.1)	59.8 (0.2)	54.9 (0.3)	63.7 (0.3)
	Reference	43.3 (0.1)	59.8 (0.2)	56.2 (0.2)	65.5 (0.4)
	Placebo	43.4 (0.1)	59.8 (0.2)	56.1 (0.2)	68.9 (0.5)

**Table 3:** Differences between treatments at 2 and 4 h post-treatment (mITT population; *n* = 62)

Time- point	Treatment comparison	Differences between treatments (adjusted mean with 95% CI); ***p***-value^**a,b**^			
		%SMHR	%RER	EFU (μg F/cm^**2**^)	ARR
2 h	Test vs Placebo	5.62 (2.80, 8.43) **0.0001**	36.41 (31.65, 41.18) **< 0.0001**	1.65 (1.41, 1.88) **< 0.0001**	0.31 (0.26, 0.35) **< 0.0001**
	Test vs Ref	3.90 (1.09, 6.72) **0.0070**	12.60 (7.84, 17.36) **< 0.0001**	0.95 (0.72, 1.19) **< 0.0001**	0.09 (0.04, 0.13) **0.0002**
	Ref vs Placebo	1.71 (−1.10, 4.53) 0.2303	23.8 (19.05, 28.58) **< 0.0001**	0.69 (0.46, 0.93) **< 0.0001**	0.22 (0.18, 0.27) **< 0.0001**
4 h	Test vs Placebo	7.69 (5.18 10.19) **< 0.0001**	33.29 (28.89, 37.68) **< 0.0001**	1.81 (1.59, 2.04) **< 0.0001**	0.26 (0.21, 0.30) **< 0.0001**
	Test vs Ref	7.57 (5.07, 10.07) **< 0.0001**	10.98 (6.58, 15.37) **< 0.0001**	0.97 (0.75, 1.20) **< 0.0001**	0.03 (−0.01, 0.08) 0.1071
	Ref vs Placebo	0.12 (−2.38, 2.62) 0.9259	22.3 (17.92, 26.70) **< 0.0001**	0.84 (0.62, 1.06) **< 0.0001**	0.22 (0.18, 0.26) **< 0.0001**

^a^From ANOVA model with fixed factors for study period and treatment, and a random effect for participant.

^b^Difference is first-named treatment minus second-named treatment, a positive difference favours first-named treatment.

Statistically significant comparisons are highlighted in bold.

Ref: Reference toothpaste.

#### Surface microhardness recovery

**Fig. 2:**
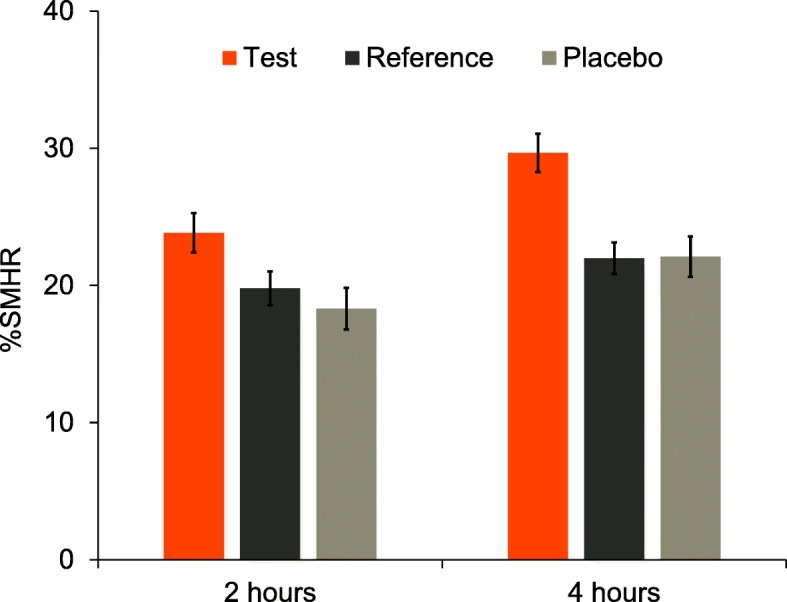
Raw mean (±SE) percent surface microhardness recovery (%SMHR) by treatment group (mITT population). Higher values are favourable

Raw mean %SMHR (±SE) is shown in Fig. 2. After 2 and 4 h, the adjusted mean %SMHR was statistically significantly greater for the Test toothpaste than for either the Placebo or Reference toothpastes (Table 3). The significant difference after 4 h met the pre-designated primary objective of the study. *Post-hoc* analysis showed no statistically significant differences between Placebo and Reference toothpastes at either timepoint (Table 3).

#### Relative erosion resistance

**Fig. 3:**
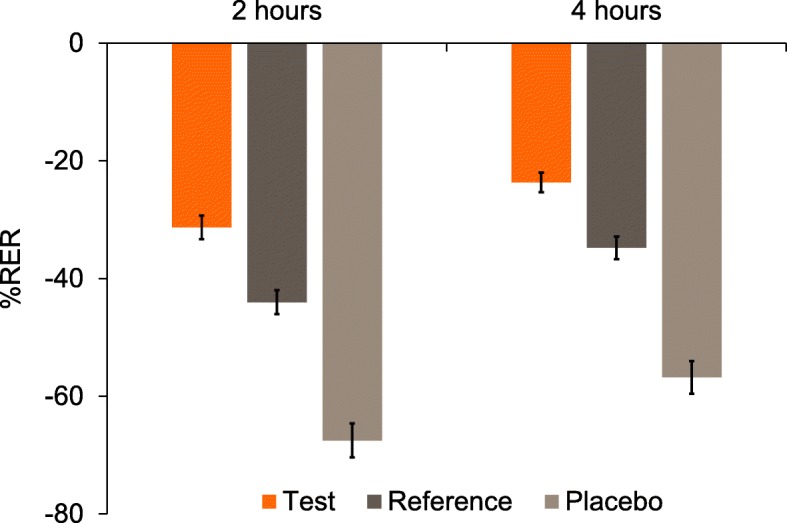
Raw mean (±SE) percent relative erosion resistance (%RER) by treatment group (mITT population). Higher (less negative) values are favourable

Raw mean %RER (±SE) is shown in Fig. 3. After 2 and 4 h, the adjusted mean %RER was statistically significantly greater (less negative) for the Test toothpaste than for either the Placebo or Reference toothpastes (Table 3). *Post-hoc* analysis showed that the %RER for the Reference toothpaste was superior to the Placebo toothpaste at both timepoints (Table 3).

#### Enamel fluoride uptake

**Fig. 4:**
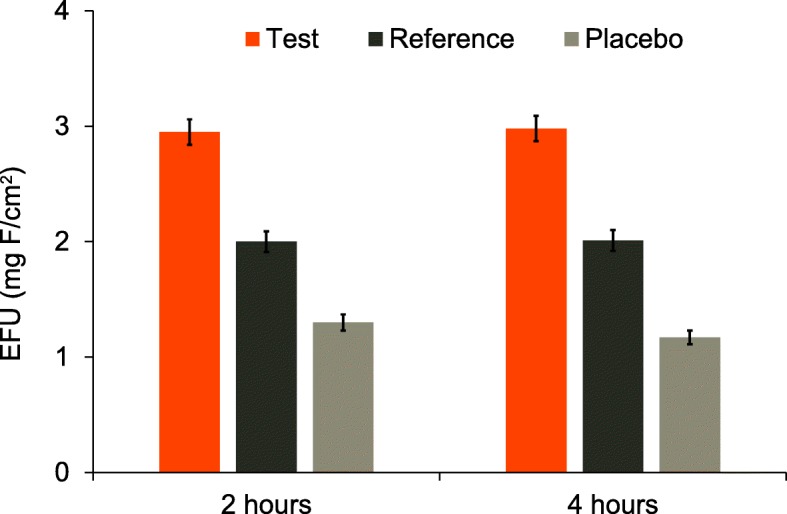
Raw mean (±SE) enamel fluoride uptake (EFU) by treatment group (mITT population). Higher values are favourable

Mean EFU (±SE) is shown in Fig. 4. After 2 and 4 h, the adjusted mean EFU was statistically significantly greater for the Test toothpaste than for either the Placebo or Reference toothpastes (Table 3). *Post-hoc* analysis showed that the EFU for the Reference toothpaste was superior to the Placebo toothpaste at both timepoints (Table 3).

#### Acid resistance ratio

**Fig. 5:**
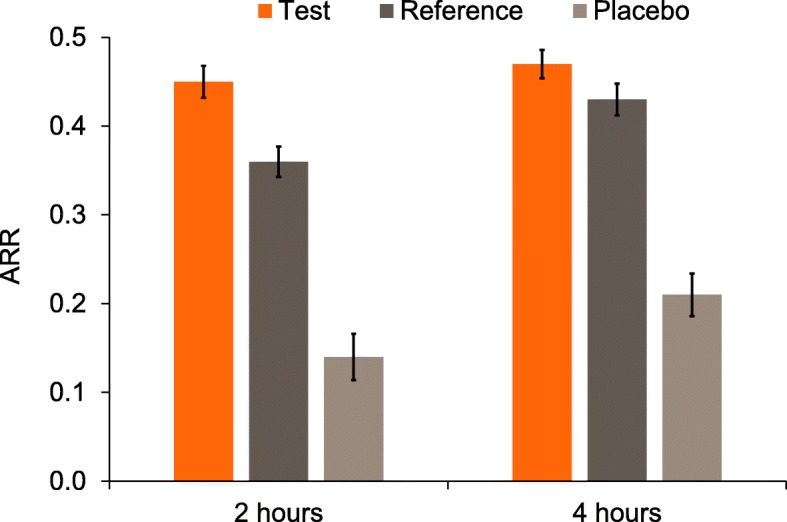
Raw mean (±SE) acid resistance ratio (ARR) by treatment group (mITT population). Higher values are favourable

Mean ARR (±SE) is shown in Fig. 5. *Post-hoc* analysis showed that after 2 and 4 h, adjusted mean ARR was statistically significantly greater for the Test than for the Placebo toothpaste (Table 3). The ARR value for the Test toothpaste was statistically significantly greater than the Reference toothpaste at 2 h, but the difference was not significant at 4 h. *Post-hoc* analysis showed that the ARR for the Reference toothpaste was superior to the Placebo toothpaste at both timepoints (Table 3).

### Safety

Twenty-one participants (33.9%) reported at least one treatment-emergent adverse event (TEAE), with 26 TEAEs in total. Twelve participants (19.4%) reported at least one oral TEAE (a total of 14 oral TEAEs), in roughly equal numbers across groups. Ten participants (16.1%) reporting at least one non-oral TEAE (12 TEAEs in total). All TEAEs were of mild or moderate intensity, resolved by study completion and did not lead to withdrawal from the study; none was considered treatment-related. There were no serious AEs reported during the study.

## Discussion

Measuring enamel erosion in vivo is technically very challenging. Changes occur on a micrometre scale (unless timescales are very long) on complex dental surfaces without stable reference points [2]. Studies with in situ models are therefore widely accepted as the current industry-standard approach to assessing treatment effects on acid erosion processes. This approach does have the limitation that such models cannot fully mimic the clinical situation; however, because enamel surfaces for in situ studies can be prepared in advance and changes measured outside of the mouth while treatment and remineralisation occur in the mouth, measurements are relatively precise, control of treatment conditions is high, and measurable changes can occur on short time-scales [3, 6–8, 11–17, 20, 21, 23, 24].

The present in situ model was designed to monitor erosive demineralisation and remineralisation processes representing typical daily behaviour. Many individuals consume an acidic beverage at breakfast, such as orange or grapefruit juice, which may soften the enamel surface (first stage of dental erosion). They may then brush with a fluoridated toothpaste, which supplies fluoride for several hours to enhance saliva-mediated rehardening of the softened enamel. This may be followed by an additional dietary erosive challenge from an acidic drink some hours later. The aim of the model is therefore to mimic such a scenario, modelling the very earliest stages of erosive demineralisation, followed by fluoride-enhanced rehardening and a subsequent acid challenge.

In the present study, our results clearly showed that the Test toothpaste – containing NaF – outperformed the Reference toothpaste – containing SnF_2_ and zinc citrate – and the fluoride-free Placebo toothpaste in terms of enamel rehardening. They also showed that the NaF Test toothpaste provided superior overall protection of the enamel surface through a cycle of treatment-induced rehardening and acid-induced demineralisation (RER). The specific measure of enamel acid resistance post-rehardening, ARR, showed superiority of the NaF toothpaste at the 2 h timepoint; this difference was no longer statistically significant at the 4 h timepoint (*p* = 0.1071). It seems that the high acid resistance after rehardening (relative to before treatment) for the Test toothpaste group is due primarily to the high level of fluoride incorporated into the newly-formed mineral. The superior EFU of the Test toothpaste treatment compared to the other two treatments supports these differences in mineralisation measures.

The low uptake of fluoride from the reference SnF_2_-Zn toothpaste, and absence of rehardening over-and-above the placebo in this type of model, is consistent with in vitro observations that stannous ions can inhibit enamel remineralisation [26, 27]. The relatively high resistance to acid, in spite of the low fluoride uptake, also suggests stannous ions inhibited enamel demineralisation, in addition to the inhibition provided by fluoride in this study [11–14].

The modes of action of the key extra ingredients of the NaF formulation, i.e., lactate ion and PVM/MA copolymer at pH 6.2, are not yet fully understood. Certain carboxylic acid polymers have been shown in vitro to enhance resistance to demineralisation [28]. It is assumed that multi-point attachment of polycarboxylic acid polymer chains, such as PVM/MA, tends to stabilise the surface against demineralisation challenges. Reducing pH from the neutral range typical of conventional dentifrices is well-established to increase EFU from fluoride products in vitro [29, 30]. At pH 6.2, in the presence of lactate ion, higher EFU has been observed in vitro compared to a neutral, lactate-free equivalent formulation (data on file). It should be noted that specific effects of these agents in a clinical situation have yet to be demonstrated.

## Conclusion

Within the limitations imposed by this short-term, in situ dental erosion rehardening model, a NaF dentifrice containing PVM/MA copolymer and lactate ion at pH 6.2 provided enhanced enamel rehardening and greater overall protection from subsequent in vitro enamel demineralisation, compared with a fluoride-free toothpaste and a marketed anti-erosion toothpaste. While the performance of the novel Test dentifrice in this model has been demonstrated, the mode of action of the ingredients in the formulation have not been established and this area warrants further research.

## Data Availability

Anonymised individual participant data and study documents can be requested for further research from www.clinicalstudydatarequest.com.

## Abbreviations

AE: Adverse event
ANOVA: Analysis of variance
ARR: Acid resistance ratio
CI: Confidence interval
EFU: Enamel fluoride uptake
HClO_4_: Perchloric acid
IRB: Institutional Review Board
KNO_3_: Potassium nitrate
mITT: Modified intent-to-treat
NaF: Sodium fluoride
OHT: Oral hard tissue
OST: Oral soft tissue
PVM/MA: Polyvinylmethylether-maleic anhydride copolymer
RER: Relative erosion resistance
SD: Standard deviation
SE: Standard error
SMH: Surface microhardness
SMHR: Surface microhardness recovery
SnF_2_: Stannous fluoride
TEAE: Treatment-emergent adverse event
Zn: Zinc
